# Optimization of agronomic practices for enhanced fodder quality and productivity of barnyard millet at Zonal Agricultural Research Station, Bengaluru, Karnataka, India

**DOI:** 10.3389/fpls.2026.1940671

**Published:** 2026-09-09

**Authors:** T. S. Sukanya, C. Rajesh, Anand G. Manegar, B. C. Umashankar, C. Chaithra, Rakesh D.

**Affiliations:** 1 Department of Agronomy, University of Agricultural Sciences, Gandhi Krishi Vignana Kendra (GKVK), Bengaluru, Karnataka, India; 2 Veterinary College, Bengaluru, Karnataka, India

**Keywords:** fodder, harvesting stages, nitrogen levels, planting geometry, yield

## Abstract

**Introduction:**

The fodder crisis is widening due to changing socio-economic preferences and a decline in the area under fodder cultivation. Therefore, there is an immediate need to explore the potential of dual-purpose crops for both food and fodder production. This study evaluated the effects of nitrogen levels, planting geometry, and harvesting stages on the productivity and quality of barnyard millet fodder.

**Methods:**

A field experiment was conducted at the Zonal Agricultural Research Station (ZARS), University of Agricultural Sciences (UAS), Gandhi Krishi Vignana Kendra (GKVK), Bengaluru, during August–November in 2024 and 2025. The experiment was laid out in a factorial randomized complete block design (RCBD) with three replications. The 12 treatment combinations comprised three nitrogen levels (N1: 40 kg N ha^-1^, N2: 60 kg N ha^-1^, and N3: 80 kg N ha^-1^), two planting geometries (P1: 30 × 10 cm and P2: 22.5 × 10 cm), and two harvesting stages (H1: earhead emergence and H2: physiological maturity) .

**Results:**

Application of 80 kg N ha^-1^ produced the highest green fodder yield (15,731 kg ha^-1^) and dry fodder yield (3,969 kg ha^-1^), followed by 60 kg N ha^-1^, whereas the lowest yields were recorded with 40 kg N ha^-1^. Closer spacing (22.5 × 10 cm) significantly increased biomass production, producing 14,999 kg ha^-1^ green fodder and 3,601 kg ha^-1^ dry fodder compared with 30 × 10 cm spacing. Harvesting at earhead emergence resulted in the highest green fodder yield (16,263 kg ha^-1^), whereas harvesting at physiological maturity produced the highest dry fodder yield (3,602 kg ha^-1^). Crude protein concentration was higher at earhead emergence (8.17%), while crude fiber concentration increased at physiological maturity (32.29%). Planting geometry did not significantly influence the qualitative fodder parameters.

**Discussion:**

Increasing nitrogen application from 40 to 80 kg N ha^-1^ progressively improved fodder productivity, while closer planting enhanced biomass production. Early harvesting at earhead emergence favoured crude protein concentration and green fodder yield, whereas harvesting at physiological maturity increased dry matter production and crude fiber concentration. Overall, closer spacing (22.5 × 10 cm) combined with 80 kg N ha^-1^ is recommended for improving fodder productivity, while the choice of harvesting stage can be adjusted according to the desired balance between yield and fodder quality.

## 1 Introduction

India, despite supporting the world’s largest livestock population, continues to face a substantial deficit in quality fodder supply. Current estimates indicate shortages of 11.24% for green fodder and 23.40% for dry fodder, posing a serious constraint to sustainable livestock production (Roy et al., 2019). This situation is exacerbated by increasing competition for arable land, where food grain production is prioritized, thereby limiting the area available for fodder crops (Chaudhary and Prabhu, 2014). Consequently, improving fodder productivity through efficient agronomic management, rather than area expansion, has become essential to meet the growing demand (Sukanya et al., 2022; Prajapati et al., 2023).

Barnyard millet is an important small millet cultivated for both grain and forage, particularly in environments where rice cultivation is constrained (Yabuno, 1987). It ranks second among small millets in India, with an annual production of approximately 96 thousand tonnes and a mean productivity of 758 kg ha^−1^ (Anon., 2017). The crop is primarily grown in the Himalayan mid-hills of Uttarakhand and the Deccan Plateau of Tamil Nadu, while its wild relative, *E. colona*, is often used as an emergency food and fodder source during drought years (Padulosi et al., 2009; Sukanya et al., 2025). Owing to its short growth duration, adaptability to marginal environments and tolerance to biotic and abiotic stresses, barnyard millet is widely regarded as a climate-resilient crop. Its grains are nutritionally valuable, containing appreciable levels of protein, dietary fiber and essential micronutrients such as iron and zinc (Saleh et al., 2013; Ugare et al., 2014; Sukanya et al., 2023). Among the cultivated species, *E. frumentacea* and *E. esculenta* are most commonly preferred (Sood et al., 2015).

The need to promote alternative fodder crops has intensified in view of the widening national fodder gap, estimated at 35.65% for green fodder and 10.95% for dry fodder (Anon., 2019). Climate variability, recurrent crop failures and stagnation in fodder crop area have further aggravated fodder scarcity, particularly in rainfed regions (Halli et al., 2018; Sathiya et al., 2024). Under these conditions, barnyard millet offers considerable potential as a low-input, climate-resilient fodder crop due to its rapid growth, early maturity and high biomass production. The crop can be harvested multiple times in a year, providing flexibility in fodder availability. Its straw contains higher crude protein and calcium than commonly used cereal residues such as rice and oat straw (Obara, 1936; Yabuno, 1987). In addition, barnyard millet forage is suitable for hay and silage production, supplying up to 61% total digestible nutrients. Under Indian conditions, the crop produces approximately 5 t ha^-1^ of dry fodder (Anon., 2014), and contributes substantially to regional fodder requirements—for instance, accounting for about 11.5% of total fodder demand in Uttarakhand (Singh and Singh, 2020). Harvesting at earlier stages further improves forage quality, with metabolizable energy values ranging from 8.5 to 9.5 MJ kg^-1^ (Panwar et al., 2016).

Optimizing the fodder potential of barnyard millet requires appropriate management of key agronomic factors. Planting geometry influences plant population density, canopy structure and light interception, thereby affecting biomass accumulation and fodder yield (Janani et al., 2022). Nitrogen management is equally critical, as nitrogen enhances tiller production, leaf area development, crude protein content and forage digestibility (Goron and Raizada, 2015; Shostko, 2014; Sukanya et al., 2021). Harvesting at early reproductive stages has been shown to improve nutritive value by increasing protein concentration and reducing fiber fractions (Joshi et al., 2007). However, information on the combined effects of planting geometry, nitrogen application and harvest timing on fodder yield and quality of barnyard millet under specific agro-ecological conditions remains limited.

In this context, the present study was conducted at the University of Agricultural Sciences, GKVK, Bengaluru, to evaluate the interactive effects of nitrogen levels, planting geometry and harvesting stage on fodder yield and quality of barnyard millet. The objective was to identify agronomic practices that enhance biomass production, improve forage nutritive value and increase production efficiency under the prevailing agro-climatic conditions. The findings are expected to contribute to the development of practical recommendations for improving fodder availability and supporting livestock-based production systems in dryland and resource-constrained regions.

## 2 Materials and methods

### 2.1 Experimental site

**Figure 1:**
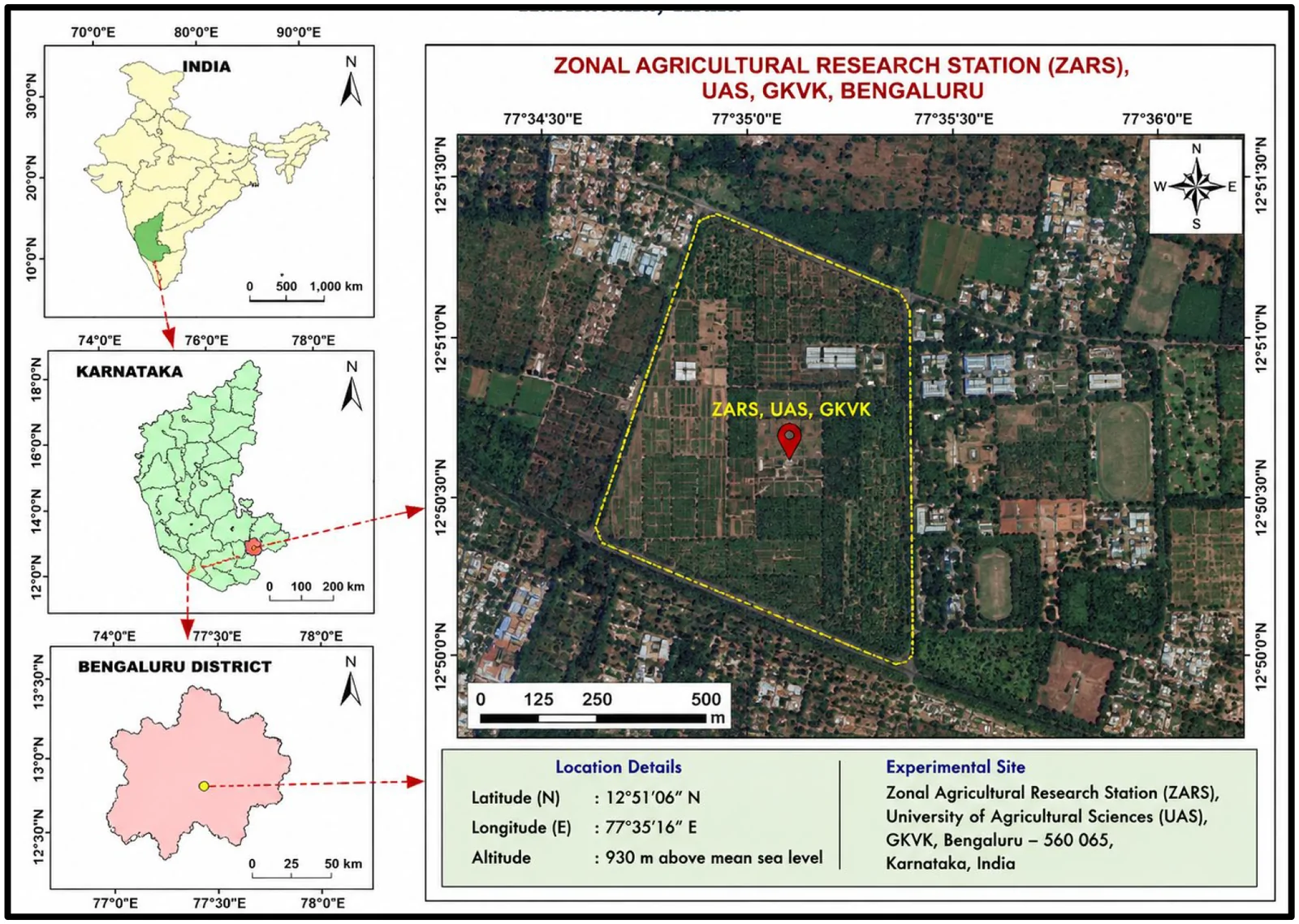
GIS-based geographical location map of the experimental site at Zonal Agricultural Research Station (ZARS), UAS, GKVK, Bengaluru, Karnataka, India.

The field experiment was conducted from August to November during 2024 and 2025 at the Zonal Agricultural Research Station, University of Agricultural Sciences, Bengaluru, Karnataka, India. The experimental site is located at a 12°51′ N latitude, 77°35′ E longitude and 930 m above MSL, under Eastern Dry Zone (ACZ- V) of Karnataka. The geographical location of the experimental site is presented in **Figure 1**. The experimental soil was classified as red sandy loam. Initial soil samples were collected before the initiation of the experiment in both years (2024 and 2025) from the plough layer (0–30 cm depth) using a soil auger of 5 cm diameter and were composited for analysis. The soil analysis revealed a slightly acidic reaction with pH values of 5.64 and 5.62 during 2024 and 2025, respectively. The electrical conductivity remained low, recording 0.22 dS m^-1^ in 2024 and 0.25 dS m^-1^ in 2025. Organic carbon content was low, ranging from 0.36% in 2024 to 0.38% in 2025. The soil was low in available nitrogen (249.7 and 252.6 kg ha^-1^ during 2024 and 2025, respectively), high in available phosphorus (46.80 and 45.12 kg ha^-1^) and medium in available potassium (180.40 and 178.20 kg ha^-1^). The soil chemical properties and analytical methods adopted are presented in **Table 1**.

**Table 1:** Soil chemical properties at the two experimental sites.

Particulars	2024	2025	References
pH (1:2: soil: water)	5.64	5.62	Potentiometric method (Piper, 1966)
EC (dS/m at 25 ^0^C)	0.22	0.25	Conductivity bridge (Jackson, 1973)
Organic carbon (%)	0.36	0.38	Walkley and Black wet oxidation method (Jackson, 1973)
Available N (kg ha^-1^)	249.7	252.6	Alkaline permanganate method (Subbiah and Asija, 1956)
Available P (kg ha^-1^)	46.80	45.12	Bray’s method (Jackson, 1973)
Available K (kg ha^-1^)	180.40	178.2	Flame photometer method (Jackson, 1973)

### 2.2 Weather conditions during the experimental period

**Figure 2:**
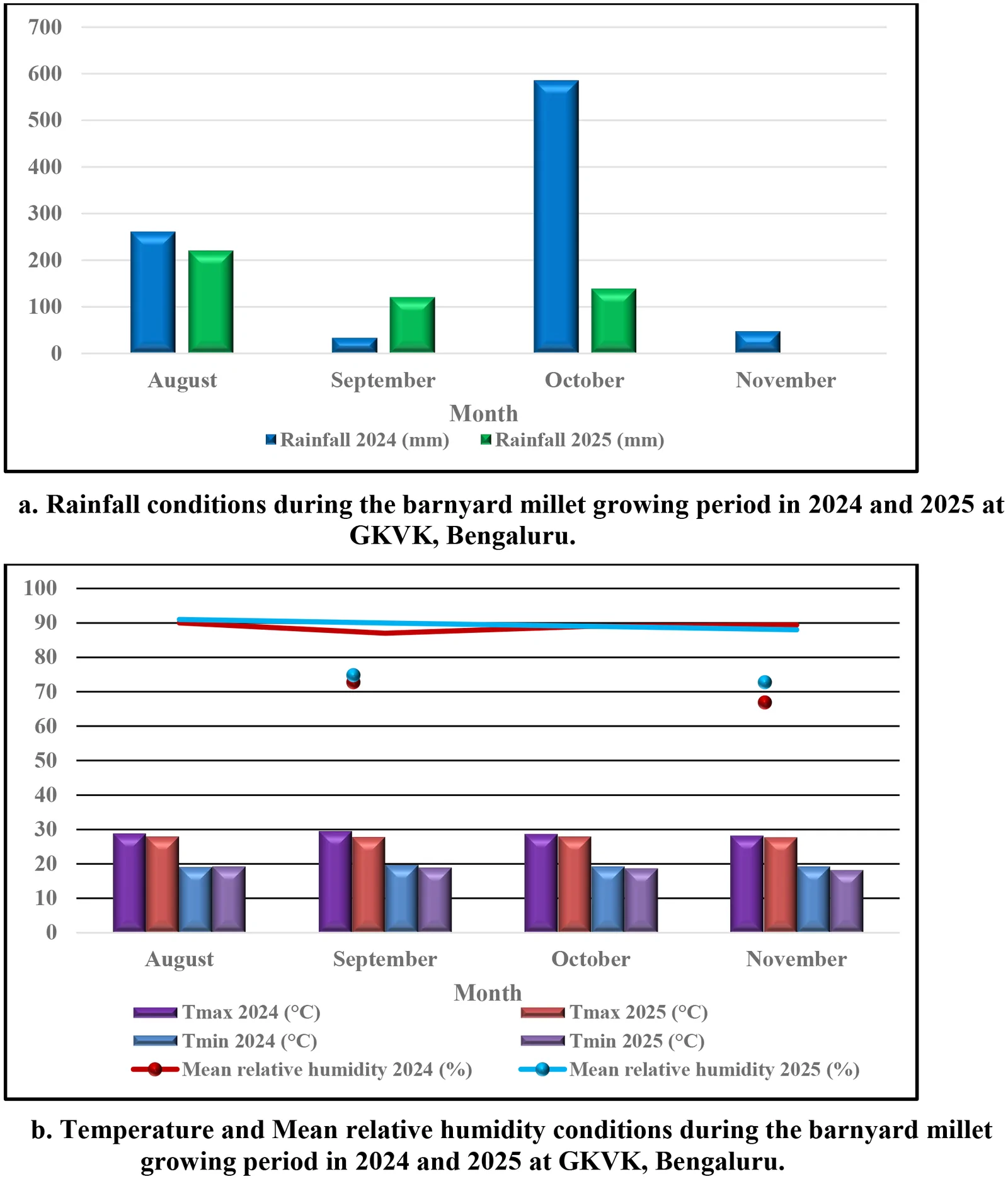
**(A)** Rainfall conditions during the barnyard millet growing period in 2024 and 2025 at GKVK, Bengaluru. **(B)** Temperature and mean relative humidity conditions during the barnyard millet growing period in 2024 and 2025 at GKVK, Bengaluru.

The meteorological conditions during the barnyard millet growing period (August–November) varied considerably between the two years, particularly with respect to rainfall distribution (**Figure 2A**). During 2024, rainfall was relatively high in August (261.2 mm) and increased markedly in October (585.4 mm), while comparatively lower rainfall was received during September (33.6 mm) and November (48.0 mm). In 2025, rainfall was more moderately distributed, with 220.0, 120.4 and 138.4 mm received during August, September and October, respectively, while no rainfall was recorded in November. The maximum temperature (**Figure 2B**) ranged from 28.1 to 29.4 °C in 2024 and from 27.7 to 27.9 °C in 2025, whereas minimum temperature varied from 19.0 to 19.6 °C and 18.1 to 19.1 °C, respectively. Mean relative humidity (**Figure 2B**) remained relatively high throughout the crop-growing period, ranging from 87.0 to 90.0 per cent in 2024 and 88.0 to 91.0 per cent in 2025. Overall, rainfall distribution showed greater year-to-year variability than temperature and relative humidity, indicating that precipitation was the major weather factor differing between the two experimental seasons. These differences in rainfall distribution and temperature conditions may have contributed to year-to-year variation in barnyard millet growth, fodder yield and nutritive quality.

### 2.3 Experimental design

**Figure 3:**
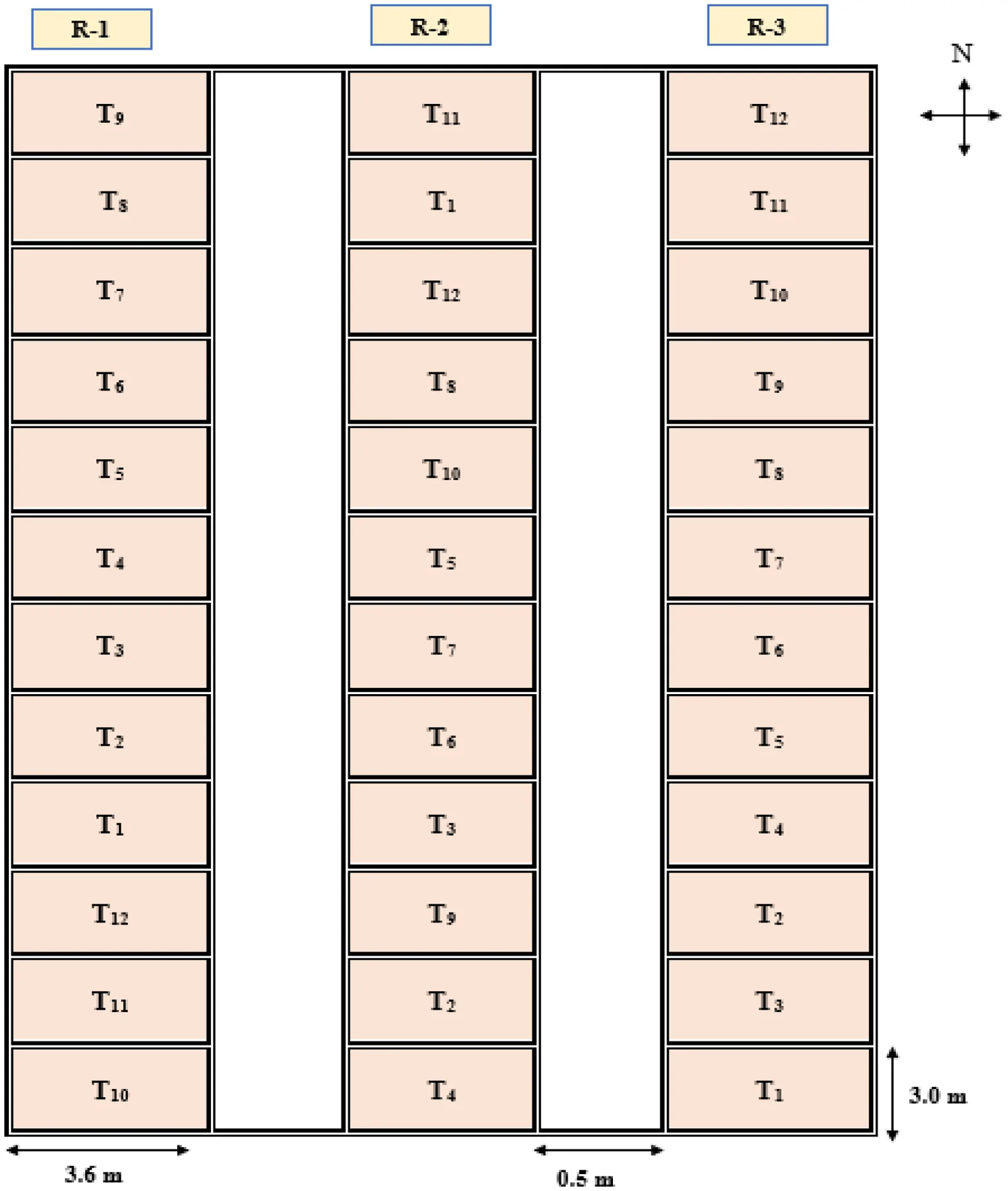
Plan of layout of the experiment plot.

The experiment was conducted using barnyard millet (*Echinochloa frumentacea* L.), variety DHBM-93-3. The experiment involved twelve treatment combinations formed by three nitrogen levels (40, 60 and 80 kg nitrogen ha^-1^), two planting geometries (30 cm × 10 cm and 22.5 cm × 10 cm) and two stages of harvest (earhead emergence and physiological maturity), laid out in a Randomized Complete Block Design under a factorial concept with three replications (**Figure 3**). Nitrogen was given in equal splits twice, half as basal at sowing and remaining quantity at 30 days after sowing, while the recommended doses of potassium and phosphorus were applied uniformly to all plots. The gross plot measured 3.6 m × 3.0 m (12.96 m^2^) and the net plot 2.4 m × 2.6 m (6.24 m^2^).

#### 2.3.1 Treatment details

T_1_- N_1_P_1_H_1–_40 kg N ha^-1^, 30 cm x 10 cm, harvest at earhead emergence

T_2_- N_1_P_1_H_2–_40 kg N ha^-1^, 30 cm x 10 cm, harvest at physiological maturity

T_3_- N_1_P_2_H_1–_40 kg N ha^-1^, 22.5 cm x 10 cm, harvest at earhead emergence

T_4_- N_1_P_2_H_2–_40 kg N ha^-1^, 22.5 cm x 10 cm, harvest at physiological maturity

T_5_- N_2_P_1_H_1–_60 kg N ha^-1^, 30 cm x 10 cm, harvest at earhead emergence

T_6_- N_2_P_1_H_2–_60 kg N ha^-1^, 30 cm x 10 cm, harvest at physiological maturity

T_7_- N_2_P_2_H_1–_60 kg N ha^-1^, 22.5 cm x 10 cm, harvest at earhead emergence

T_8_- N_2_P_2_H_2–_60 kg N ha^-1^, 22.5 cm x 10 cm, harvest at physiological maturity

T_9_- N_3_P_1_H_1–_80 kg N ha^-1^, 30 cm x 10 cm, harvest at earhead emergence

T_10_- N_3_P_1_H_2–_80 kg N ha^-1^, 30 cm x 10 cm, harvest at physiological maturity

T_11_- N_3_P_2_H_1–_80 kg N ha^-1^, 22.5 cm x 10 cm, harvest at earhead emergence

T_12_- N_3_P_2_H_2–_80 kg N ha^-1^, 22.5 cm x 10 cm, harvest at physiological maturity

### 2.4 Data collection

Morphological observations were recorded using plant samples collected from each net plot. Five plants were selected at random from each plot and used for recording growth and yield parameters at 30 and 60 days after sowing (DAS) and at harvest. The mean of observations recorded from the five plants was used for statistical analysis.

### 2.5 Growth parameters

Five representative plants from each net plot were randomly selected, tagged, and used for recording growth observations at 30 DAS, 60 DAS and at harvest.

#### 2.5.1 Plant height (cm)

Plant height was measured in centimeters on five randomly selected plants from each plot. Measurements were taken from the ground level to the base of the fully emerged leaf at 30 and 60 DAS. At harvest, plant height was measured from the base of the plant to the base of the ear head following ear head emergence. The mean plant height of five plants was used for analysis.

#### 2.5.2 Leaf area index

Leaf area index (LAI) was calculated following the method of Watson (1952) by dividing the total leaf area per plant by the corresponding ground area occupied by the plant, using the formula:


$$\text{Leaf area index}\left(\text{LAI}\right)=\frac{\text{Leaf area per plant}\left({\text{cm}}^{2}\right)}{\text{Ground area per plant}\left({\text{cm}}^{2}\right)}$$

#### 2.5.3 Leaf to stem ratio (g g^-1^)

Leaf to stem ratio was determined by separating the leaf and stem components of the sampled plants. The separated plant parts were oven-dried to constant weight, and their dry weights were recorded. The leaf to stem ratio was calculated by dividing the leaf dry weight by the stem dry weight and expressed on a dry weight basis.

### 2.6 Yield measurements

#### 2.6.1 Green fodder yield (kg ha^-1^)

Green fodder yield was determined by harvesting the entire net plot area at the designated harvest stages earhead emergence and physiological maturity. Fresh biomass from each plot was weighed immediately after harvest and converted to yield per hectare.

#### 2.6.2 Dry fodder yield (kg ha^-1^)

A representative subsample of fresh fodder from each treatment was oven-dried at 70 ± 5 °C to a constant weight. The dry mass was used to calculate dry fodder yield per hectare by extrapolating the sample dry weight to the total fresh biomass.

### 2.7 Soil nutrient analysis

#### 2.7.1 Soil sampling and processing

Composite soil samples (0–30 cm depth) were collected before sowing and immediately after harvest to assess the initial soil characteristics and nutrient changes. Samples were air-dried, crushed, and sieved through a 2-mm sieve for routine analysis. For organic carbon, samples were further ground and passed through a 0.2-mm sieve.

### 2.8 Plant nutrient analysis

#### 2.8.1 Collection and preparation of plant samples

Post-harvest grain and straw samples were oven-dried at 65 °C, finely ground, and stored for nutrient analysis. Nitrogen, phosphorus, and potassium contents were estimated following standard procedures.

#### 2.8.2 Nitrogen estimation (%)

Nitrogen content was analyzed using the micro-Kjeldahl method (Jackson, 1973). A 0.5 g ground sample was digested in concentrated H_2_SO_4_ with a digestion mixture (K_2_SO_4_:CuSO_4_·5H_2_O:Selenium = 100:20:1), distilled under alkaline conditions, and titrated against standard H_2_SO_4_. Nitrogen content was expressed on a percentage basis.

#### 2.8.3 Di-acid digestion of plant samples

A 0.5 g powdered sample was pre-digested with 5 ml concentrated HNO_3_, then digested using aHNO_3_:HClO_4_ (9:4) mixture (Jackson, 1973). The digest was diluted to 100 ml and used for determining all nutrients except nitrogen.

#### 2.8.4 Phosphorus estimation (%)

Phosphorus was determined colorimetrically using the vanado-molybdate yellow color method (Jackson, 1973) from an aliquot of the di-acid digest.

#### 2.8.5 Potassium estimation (%)

Potassium concentration in the digest was quantified using a calibrated flame photometer, following standard procedures (Jackson, 1973).

#### 2.8.6 Nutrient uptake (kg ha^-1^)

Nutrient uptake was calculated using dry matter yield and the corresponding nutrient concentration, as:


$$\text{Nutrient uptake}\left({\text{kg ha}}^{-1}\right)=\frac{\%\text{ Nutrient content}\times \text{Dry matter yield}\left({\text{kg ha}}^{-1}\right)}{100}$$

### 2.9 Biochemical analysis of fodder quality

Treatment-wise straw samples were ground and analyzed for crude protein, crude fiber, ether extract, ash, ADF, NDF and lignin using standard laboratory procedures.

#### 2.9.1 Crude protein (%)

Nitrogen content, determined by the Kjeldahl method (Jackson, 1973), was multiplied by 6.25 to derive crude protein content.

#### 2.9.2 Crude fiber (%)

Crude fiber was determined following Anon. (1977). A 5 g moisture- and fat-free sample was sequentially digested with 0.25 N H_2_SO_4_ and 1.25% NaOH, filtered, washed, oven-dried (80–100 °C), and finally ashed at 600 °C. Fiber content was calculated from the difference in weights.

#### 2.9.3 Ether extractable fat (%)

Fat content was estimated using the Soxhlet extraction method with petroleum ether. The extracted fat residue was weighed and expressed as a percentage of oven-dry sample weight.

#### 2.9.4 Ash content (%)

Ash content was determined by igniting an oven-dried sample at 550 ± 25 °C for 4–6 hours in a muffle furnace and expressing the mineral residue as a percentage of sample dry weight.

#### 2.9.5 Acid detergent fiber (%)

ADF was estimated following Goering and Van Soest (1975). One gram of dried sample was refluxed for 1 h in acid detergent solution with decahydronaphthalene, filtered, washed (hot water, acetone, hexane), dried, and weighed. ADF (%) was calculated as:


$$\text{ADF}\left(\%\right)=\frac{\left({\text{W}}_{1}-{\text{W}}_{0}\right)}{\text{Weight of oven dry sample taken}\left(\text{g}\right)}\times 100$$

#### 2.9.6 Neutral detergent fiber (%)

NDF content was estimated using the Goering and Van Soest (1975) procedure. Samples were refluxed in neutral detergent solution with sodium sulphite and decahydronaphthalene, filtered, washed, dried, and weighed. NDF (%) was derived as:


$$\text{NDF}\left(\%\right)=\frac{\left({\text{W}}_{1}-{\text{W}}_{0}\right)}{\text{Weight of oven dry sample taken}\left(\text{g}\right)}\times 100$$

#### 2.9.7 Lignin content (%)

Lignin was quantified using the acid detergent lignin (ADL) method (Goering and Van Soest, 1975). The ADF residue was treated with 72% H_2_SO_4_, washed, dried, ashed and lignin content was calculated as:


$$\text{Lignin content}\left(\%\right)=\frac{\text{ADL residue weight}\left(\text{g}\right)-\text{Ash weight}\left(\text{g}\right)}{\text{sample weight}\left(\text{g}\right)}\times 100$$

#### 2.9.8 Dry matter digestibility (%)

Dry matter digestibility was estimated indirectly from the acid detergent fiber (ADF) content using the standard predictive equation proposed by Harris et al. (1970) and commonly used in forage quality evaluation. The following regression equation was applied:


DMD(%)=88.9−(0.779×%ADF, dry matter basis)

#### 2.9.9 Total digestible nutrients (%)

Total digestible nutrients were predicted from ADF values using the regression equation recommended by Holland (1990) for tropical forages:

TDN(%)=(−1.291×ADF%)+101.35.

ADF (%) obtained from fiber analysis was used to compute TDN for each treatment.

#### 2.9.10 Nitrogen free extract (%)

Nitrogen free extract, representing the readily available carbohydrate fraction of the fodder, was calculated following the proximate analysis procedure of AOAC (2019). After estimating crude protein (CP), crude fiber (CF), ether extract (crude fat), and ash content on a dry-matter basis, NFE was obtained by difference using the formula:


NFE(%)=100−[CP(%)+Crude fat(%)+CF(%)+Ash(%)]

#### 2.9.11 Estimation of cell content (%)

Cell content, representing the non-fiber portion of the forage, was calculated from neutral detergent fiber (NDF) values using the method suggested by Goering and Van Soest (1975):


Cell content(%)=100−NDF(%)

### 2.10 Statistical analysis

The data recorded during the experiment data were analyzed using ANOVA - Analysis of Variance technique appropriate for a three-factor factorial experiment in a randomized complete block design (RCBD), as outlined by Gomez and Gomez (1984). The effects of nitrogen level (N), planting geometry (P), harvesting stage (H) and their interactions (N × P, N × H, P × H and N × P × H) were tested. The factorial arrangement of treatments allowed evaluation of individual main effects as well as their interactions, while blocking minimized the variability due to field heterogeneity. The significance of treatment effects was tested at the probability level of 5%. Wherever significant effects were shown, critical difference (C.D.) values were determined for comparing treatment means.

## 3 Results and discussion

### 3.1 Plant growth parameters

Nitrogen levels significantly influenced plant height, leaf area index (LAI) and leaf to stem ratio during both years of study and in the pooled analysis (**Table 2**). Increasing nitrogen application resulted in a progressive increase in plant height, with 80 kg N ha^-1^ (N_3_) producing the tallest plants (107.7 cm pooled), followed by 60 kg N ha^-1^ (97.6 cm), while the lowest values were recorded with 40 kg N ha^-1^ (81.5 cm). A similar trend was observed for LAI and leaf to stem ratio, where N_3_ registered the highest pooled LAI (1.88) and leaf to stem ratio (0.64).

**Table 2:** Effect of nitrogen levels, planting geometry and harvesting stages on plant height (cm), Leaf area index and leaf to stem ratio of barnyard millet (grouped data of two years).

Treatment	Plant height			Leaf area index			Leaf to stem ratio		
	2024	2025	Pooled	2024	2025	Pooled	2024	2025	Pooled
Nitrogen levels (N)									
N_1–_40 kg N ha^-1^	82.2	80.5	81.5	1.21	1.22	1.22	0.28	0.31	0.31
N_2–_60 kg N ha^-1^	97.5	97.5	97.6	1.52	1.60	1.56	0.57	0.57	0.57
N_3–_80 kg N ha^-1^	107.7	107.6	107.7	1.86	1.90	1.88	0.63	0.65	0.64
S. Em. ±	2.49	2.75	2.67	0.06	0.04	0.03	0.02	0.02	0.01
C.D. (P = 0.05)	7.29	8.06	7.82	0.19	0.12	0.09	0.07	0.05	0.04
Planting geometry (P)									
P_1–_30 cm x 10 cm	92.7	91.4	92.1	1.43	1.48	1.45	0.52	0.53	0.52
P_2_**-** 22.5 cm x 10 cm	98.9	99.0	99.0	1.63	1.67	1.65	0.46	0.49	0.49
S. Em. ±	2.03	2.24	2.18	0.05	0.03	0.03	0.02	0.01	0.01
C.D. (P = 0.05)	5.95	6.58	6.39	0.15	0.10	0.08	0.06	0.04	0.03
Harvesting stages (H)									
H_1_ **-** Earhead emergence	92.4	91.7	92.2	1.91	1.94	1.92	0.61	0.64	0.64
H_2_ **-** Physiological maturity	99.2	98.7	99.0	1.15	1.21	1.18	0.37	0.37	0.37
S. Em. ±	2.03	2.24	2.18	0.05	0.03	0.03	0.02	0.01	0.01
C.D. (P = 0.05)	5.95	6.58	6.39	0.15	0.10	0.08	0.06	0.04	0.03
Interaction (N x P)									
S. Em. ±	3.51	3.45	3.46	0.09	0.07	0.06	0.03	0.02	0.03
C.D. (P = 0.05)	NS	NS	NS	NS	NS	NS	NS	NS	NS
Interaction (N x H)									
S. Em. ±	3.51	3.45	3.46	0.09	0.07	0.06	0.03	0.02	0.03
C.D. (P = 0.05)	NS	NS	NS	NS	NS	NS	NS	NS	NS
Interaction (P x H)									
S. Em. ±	2.87	2.81	2.82	0.07	0.06	0.05	0.02	0.01	0.02
C.D. (P = 0.05)	NS	NS	NS	NS	NS	NS	NS	NS	NS
Interaction (N x P x H)									
S. Em. ±	4.97	5.50	5.33	0.13	0.08	0.06	0.05	0.03	0.03
C.D. (P = 0. 05)	NS	NS	NS	NS	NS	NS	NS	NS	NS

Planting geometry had a significant effect on all growth parameters across years and in pooled data (**Table 2**). The closer spacing of 22.5 cm × 10 cm (P_2_) resulted in significantly higher pooled plant height (99.0 cm) and LAI (1.65) compared with the wider spacing of 30 cm × 10 cm (P_1_). Conversely, the leaf to stem ratio was significantly higher under P_1_ (0.52 pooled) than under P_2_ (0.49 pooled).

Harvesting stage significantly affected plant height, LAI and leaf to stem ratio. Plants harvested at physiological maturity (H_2_) recorded significantly greater plant height (99.0 cm pooled) than those harvested at earhead emergence (H_1_) (92.2 cm pooled). In contrast, LAI and leaf to stem ratio were significantly higher at earhead emergence, with pooled values of 1.92 and 0.64, respectively, compared with 1.18 and 0.37 at physiological maturity.

The two-way and three-way interactions among nitrogen levels, planting geometry, and harvesting stages were non-significant for plant height, leaf area index (LAI), and leaf-to-stem ratio in both years as well as in the pooled analysis (**Table 2**), indicating that the effect of each factor on these growth and quality parameters was largely independent of the other factors.

### 3.2 Fodder yield

Nitrogen levels significantly affected green fodder yield and dry fodder yield during both years of experimentation and in the pooled analysis (**Table 3**). Application of 80 kg N ha^-1^ (N_3_) produced the highest pooled green fodder yield (15669 kg ha^-1^), which was significantly superior to 60 kg N ha^-1^ (14590 kg ha^-1^) and 40 kg N ha^-1^ (12790 kg ha^-1^). A similar trend was observed for dry fodder yield, with N_3_ recording the maximum pooled yield (3911 kg ha^-1^), followed by N_2_ (3685 kg ha^-1^), while the lowest yield was obtained under N_1_ (2689 kg ha^-1^).

**Table 3:** Effect of nitrogen levels, planting geometry and harvesting stages on green and dry fodder yield of barnyard millet (grouped data of two years).

Treatment	Green fodder yield (kg ha^-1^)			Dry fodder yield (kg ha^-1^)		
	2024	2025	Pooled	2024	2025	Pooled
Nitrogen levels (N)						
N_1–_40 kg N ha^-1^	13123	12521	12790	2731	2655	2689
N_2–_60 kg N ha^-1^	14679	14582	14590	3670	3706	3685
N_3–_80 kg N ha^-1^	15731	15650	15669	3969	3832	3911
S. Em. ±	417	476	407	102	107	99
C.D. (P = 0.05)	1223	1395	1195	300	314	290
Planting geometry (P)						
P_1–_30 cm x 10 cm	14000	13680	13855	3313	3265	3270
P_2_ **-** 22.5 cm x 10 cm	14999	14821	14844	3601	3531	3586
S. Em. ±	340	388	333	84	87	81
C.D. (P = 0.05)	998	1139	976	245	257	237
Harvesting stages (H)						
H_1_ **-** Earhead emergence	16263	16152	16174	3312	3269	3306
H_2_ **-** Physiological maturity	12760	12349	12526	3602	3526	3551
S. Em. ±	340	388	333	84	87	81
C.D. (P = 0.05)	998	1139	976	245	257	237
Interaction (N x P)						
S. Em. ±	490	485	486	114	108	112
C.D. (P = 0.05)	NS	NS	NS	NS	NS	NS
Interaction (N x H)						
S. Em. ±	490	485	486	114	108	112
C.D. (P = 0.05)	NS	NS	NS	NS	NS	NS
Interaction (P x H)						
S. Em. ±	400	396	397	93	88	92
C.D. (P = 0.05)	NS	NS	NS	NS	NS	NS
Interaction (N x P x H)						
S. Em. ±	834	951	815	205	214	198
C.D. (P = 0.05)	NS	NS	NS	NS	NS	NS

Planting geometry exerted a significant influence on both green and dry fodder yields (**Table 3**). The closer spacing of 22.5 cm × 10 cm (P_2_) resulted in significantly higher pooled green fodder yield (14844 kg ha^-1^) and dry fodder yield (3586 kg ha^-1^) compared with the wider spacing of 30 cm × 10 cm (P_1_), which recorded pooled yields of 13855 kg ha^-1^ and 3270 kg ha^-1^, respectively. Closer spacing likely facilitated more efficient utilization of available space, greater canopy coverage and better interception of solar radiation per unit area, ultimately enhancing total biomass productivity. This is in conformity with the findings of Shekara et al. (2019) and Kale and Takawale (2023), in fodder pearl millet.

Harvesting stages also had a significant effect on fodder yield. Harvesting at earhead emergence (H_1_) produced significantly higher pooled green fodder yield (16174 kg ha^-1^) than harvesting at physiological maturity (H_2_) (12526 kg ha^-1^). In contrast, dry fodder yield was significantly higher at physiological maturity (3551 kg ha^-1^ pooled) compared with earhead emergence (3306 kg ha^-1^). This differential response between green and dry fodder aligns with the physiological changes occurring during crop development, where fresh weight declines but dry matter proportion increases with advancing maturity. The absence of significant interaction among nitrogen, spacing and harvesting stage indicates that each factor influenced fodder productivity independently, suggesting that the physiological response of barnyard millet to nutrient availability, plant density and maturity stage does not substantially overlap.

The interaction effect among nitrogen levels and planting geometry, nitrogen levels and harvesting stages, planting geometry and harvesting stages and nitrogen levels, planting geometry and harvesting stages (N × P × H) was not significant for green fodder yield and dry fodder yield during both years and in the pooled analysis (**Table 3**).

### 3.3 Nutrient uptake

**Figure 4:**
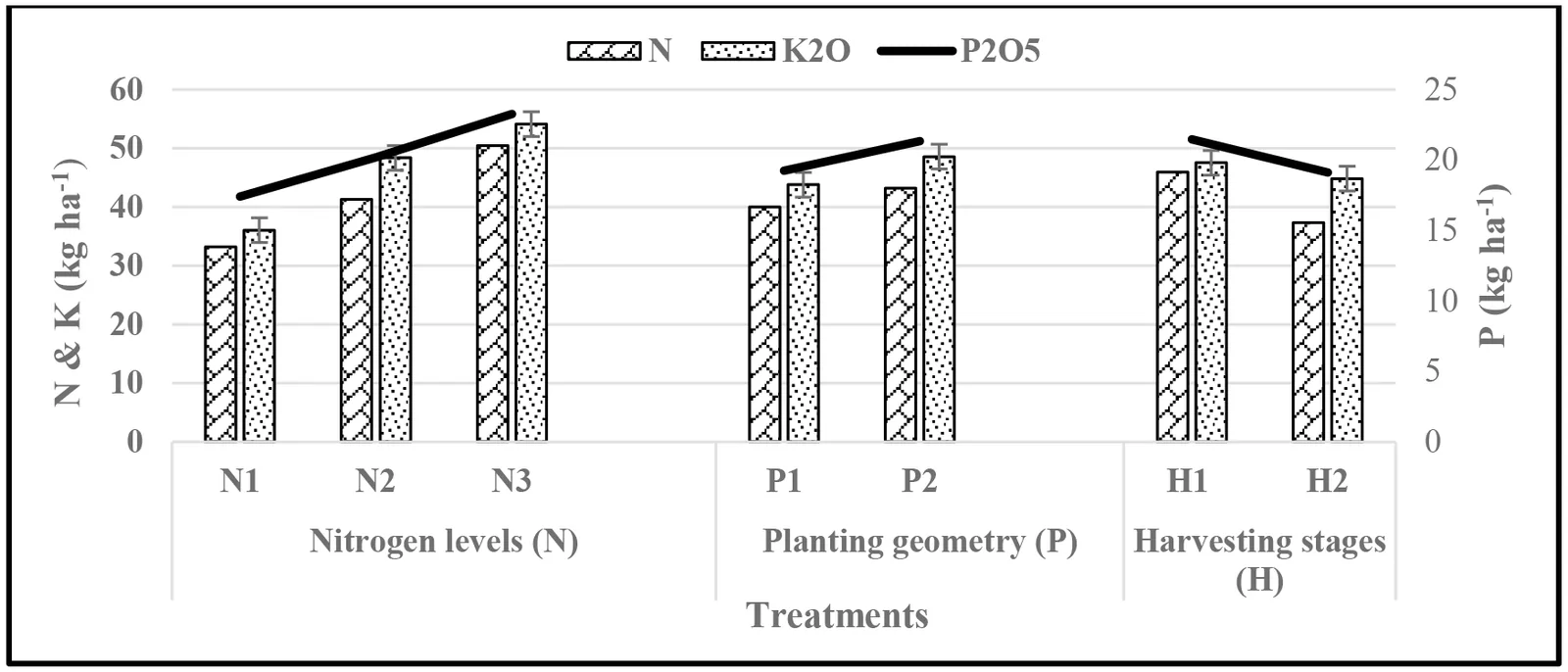
Effect of nitrogen levels, planting geometry and harvest stages on uptake of N, P_2_O_5_ and K_2_O in barnyard millet (grouped data of two years).

The uptake of N, P_2_O_5_ and K_2_O by barnyard millet differed markedly among nitrogen levels, planting geometry and harvesting stages (**Figure 4**). A clear upward trend in nutrient uptake was observed with increasing nitrogen application, with N_3_ (80 kg N ha^-1^) recording the highest uptake of both N and K_2_O (approximately 50–55 kg ha^-1^) and P_2_O_5_ uptake nearing 20 kg ha^-1^. This progressive increase from N_1_ → N_2_ → N_3_ indicates that greater nitrogen availability stimulates vigorous vegetative growth, enhances root surface area and increases enzymatic activity linked to nutrient assimilation. Such responses are consistent with earlier forage research showing that higher nitrogen supply accelerates nutrient transport and biomass accumulation in cereal forages. The lower nutrient removal under N_1_ further highlights the limitations in physiological activity when nitrogen is insufficient. This result is similar to the findings of Basavarajappa et al. (2002) and Jyothi et al. (2015) in foxtail millet.

Planting geometry had a noticeable impact on nutrient uptake, with the closer spacing (P_2_: 22.5 cm × 10 cm) consistently outperforming the wider spacing (P_1_). Greater uptake under P_2_ is attributed to higher plant population density, which increases collective nutrient demand and improves root competition, thereby intensifying nutrient scavenging from the soil. This observation aligns with reports in other small millets and fodder grasses, where moderate increases in population density enhanced nutrient-use efficiency without compromising individual plant performance. These results are in close conformity with the findings of Kumar et al. (2019) and Jawahar et al. (2021) in finger millet.

Nutrient uptake also varied across harvesting stages. Ear head emergence (H_1_) recorded higher uptake of N and K_2_O relative to physiological maturity (H_2_), reflecting the crop’s heightened metabolic activity and rapid nutrient translocation during early reproductive development. As the crop reached maturity, nutrient uptake declined because root activity slows and the plant relies more on internal remobilization of previously absorbed nutrients. Potassium uptake remained consistently higher than N and P_2_O_5_ across all treatments, underscoring its critical role in maintaining turgor pressure, stomatal regulation and cell wall development functions that are especially important in forage grasses known for rapid biomass accumulation. Collectively, the results illustrate that nutrient uptake in barnyard millet was associated with nitrogen availability, plant density and physiological stage of growth. Higher nitrogen application, optimum planting geometry and timely harvesting not only enhance nutrient assimilation but also contribute to improved fodder yield and quality. These findings emphasize the need for integrated nutrient and crop management strategies to optimize nutrient-use efficiency and sustain long-term soil fertility in forage-based production systems (Khinchi et al., 2017 in pearl millet).

Note: Error bars indicate standard deviation (SD). Nitrogen levels: N_1–_40 kg N ha^-1^, N_2–_60 kg N ha^-1^, N_3–_80 kg N ha^-1^; Planting geometry: P_1–_30 cm x 10 cm, P_2_ **–** 22.5 cm x 10 cm; Harvesting stages: H_1_ - Earhead emergence, H_2_ **-** Physiological maturity. .

### 3.4 Quality parameters

#### 3.4.1 Crude protein, crude fiber, ash content

Crude protein, crude fiber and ash content of the fodder were significantly influenced by nitrogen fertilization levels and harvesting stages across both years of experimentation and in the pooled analysis, while planting geometry and interaction effects (N × P × H) did not exert a significant influence on these quality parameters (**Table 4**).

**Table 4:** Effect of nitrogen, spacing and harvest stage on crude protein, crude fiber, ash contentin barnyard millet (grouped data of two years).

Treatment	Crude protein			Crude fiber			Ash content (%)		
	2024	2025	Pooled	2024	2025	Pooled	2024	2025	Pooled
Nitrogen levels (N)									
N_1–_40 kg N ha^-1^	6.26	5.31	5.78	32.72	31.14	31.93	11.20	11.13	11.16
N_2–_60 kg N ha^-1^	6.82	5.96	6.39	29.94	30.48	30.21	12.20	12.32	12.26
N_3–_80 kg N ha^-1^	7.30	6.97	7.14	27.04	27.28	27.16	12.54	12.47	12.49
S. Em. ±	0.15	0.15	0.15	0.91	0.27	0.45	0.11	0.09	0.07
C.D. (P = 0.05)	0.43	0.44	0.44	2.68	0.78	1.32	0.32	0.25	0.21
Planting geometry (P)									
P_1–_30 cm x 10 cm	6.83	6.09	6.46	29.30	29.32	29.31	11.91	11.92	11.91
P_2_ **-** 22.5 cm x 10 cm	6.76	6.07	6.41	30.50	29.95	30.22	12.04	12.03	12.02
S. Em. ±	0.12	0.12	0.12	0.75	0.22	0.37	0.09	0.07	0.06
C.D. (P = 0.05)	NS	NS	NS	NS	NS	NS	NS	NS	NS
Harvesting stages (H)									
H_1_ **-** Earhead emergence	8.17	7.51	7.84	27.51	27.62	27.57	12.21	12.21	12.21
H_2_ **-** Physiological maturity	5.41	4.66	5.03	32.29	31.65	31.97	11.75	11.73	11.73
S. Em. ±	0.12	0.12	0.12	0.75	0.22	0.37	0.09	0.07	0.06
C.D. (P = 0.05)	0.35	0.36	0.36	2.19	0.64	1.07	0.26	0.21	0.17
Interaction (N x P)									
S. Em. ±	0.24	0.21	0.23	0.96	0.95	0.96	0.19	0.18	0.19
C.D. (P = 0.05)	NS	NS	NS	NS	NS	NS	NS	NS	NS
Interaction (N x H)									
S. Em. ±	0.24	0.21	0.23	0.96	0.95	0.96	0.19	0.18	0.19
C.D. (P = 0.05)	NS	NS	NS	NS	NS	NS	NS	NS	NS
Interaction (P x H)									
S. Em. ±	0.19	0.18	0.19	0.79	0.79	0.79	0.12	0.11	0.12
C.D. (P = 0.05)	NS	NS	NS	NS	NS	NS	NS	NS	NS
Interaction (N x P x H)									
S. Em. ±	0.29	0.30	0.30	1.83	0.53	0.90	0.22	0.17	0.14
C.D. (P = 0.05)	NS	NS	NS	NS	NS	NS	NS	NS	NS

Crude protein content increased progressively with increasing nitrogen application. In the pooled analysis, application of 80 kg N ha^-1^ resulted in the highest crude protein content (7.14%), which was significantly superior to 60 kg N ha^-1^ (6.39%) and 40 kg N ha^-1^ (5.78%). The observed improvement in crude protein with higher nitrogen supply reflects enhanced nitrogen uptake and assimilation, leading to greater synthesis and accumulation of nitrogenous compounds in plant tissues. Similar responses of forage crops to increasing nitrogen fertilization have been widely reported, highlighting the central role of nitrogen in improving forage nutritive value.

In contrast, crude fiber content decreased significantly with increasing nitrogen levels. The lowest pooled crude fiber content (27.16%) was recorded with 80 kg N ha^-1^, whereas the highest value (31.93%) occurred under 40 kg N ha^-1^. Reduced fiber concentration under higher nitrogen regimes may be attributed to increased leaf proportion and reduced deposition of structural carbohydrates, resulting in more digestible forage.

Ash content exhibited a significant positive response to nitrogen application, with the highest pooled ash content (12.49%) observed at 80 kg N ha^-1^, followed by 60 kg N ha^-1^ (12.26%), while the lowest ash content (11.16%) was recorded at 40 kg N ha^-1^. The increase in ash content under higher nitrogen fertilization suggests improved uptake and accumulation of mineral elements in the herbage. .

Planting geometry did not significantly affect crude protein, crude fiber or ash content in either year or in the pooled analysis. Although minor numerical variations were observed, these differences were not statistically significant, indicating that the spacing treatments evaluated did not substantially alter the chemical composition of the fodder.

Harvesting stage exerted a pronounced influence on all quality attributes. Harvesting at earhead emergence resulted in significantly higher pooled crude protein content (7.84%) compared with harvesting at physiological maturity (5.03%). The higher protein content at the earlier stage can be attributed to a greater proportion of leaf tissue and higher metabolic activity, whereas advancing maturity leads to dilution of nitrogen concentration due to increased accumulation of stem biomass. Conversely, crude fiber content increased significantly with delayed harvesting, with physiological maturity recording higher pooled crude fiber content (31.97%) than earhead emergence (27.57%). This increase is associated with progressive lignification and thickening of cell walls as plants advance in maturity. Ash content was also significantly higher at earhead emergence (12.21%) compared to physiological maturity (11.73%), likely reflecting greater mineral concentration in younger plant tissues.

The interaction effects among nitrogen levels and planting geometry, nitrogen levels and harvesting stages, planting geometry and harvesting stages and nitrogen levels, planting geometry and harvesting stages were non-significant for crude protein, crude fiber and ash content, indicating that the responses to nitrogen fertilization and harvesting stage were consistent across the planting geometries tested. Overall, the results demonstrate that higher nitrogen application combined with harvesting at earhead emergence markedly improves forage quality by enhancing crude protein and mineral content while reducing crude fiber concentration, in line with the quality standards expected for high-value forage production. The results are supported by earlier research by Noor et al. (2018) in pearl millet, Nandini et al. (2018) in foxtail millet and Bhilare et al. (2010) in forage pearl millet.

#### 3.4.2 Acid detergent fiber & neutral detergent fiber

Nitrogen levels exerted a significant influence on forage quality parameters, namely acid detergent fiber (ADF), neutral detergent fiber (NDF) and lignin content, during both years of experimentation and in the pooled analysis (**Table 5**). A consistent decline in fiber fractions was observed with increasing nitrogen application. The lowest pooled ADF (24.48%), NDF (46.12%) and lignin (2.68%) contents were recorded with 80 kg N ha^-1^ (N_3_), which were significantly lower than those obtained with 60 kg N ha^-1^ (26.72%, 50.44% and 2.98%, respectively) and 40 kg N ha^-1^ (28.81%, 52.98% and 3.32%, respectively). The reduction in fiber fractions at higher nitrogen levels may be attributed to enhanced nitrogen availability promoting leaf development and increasing the proportion of cell contents relative to cell wall constituents, thereby improving forage quality.

**Table 5:** ADF, NDF and lignin composition of barnyard millet as affected by nitrogen levels, planting geometry and harvesting stages (grouped data of two years).

Treatment	ADF			NDF			Lignin		
	2024	2025	Pooled	2024	2025	Pooled	2024	2025	Pooled
Nitrogen levels (N)									
N_1–_40 kg N ha^-1^	28.83	28.78	28.81	53.09	52.87	52.98	3.32	3.32	3.32
N_2–_60 kg N ha^-1^	26.56	26.88	26.72	50.30	50.59	50.44	2.98	2.98	2.98
N_3–_80 kg N ha^-1^	24.31	24.65	24.48	45.77	46.47	46.12	2.68	2.69	2.68
S. Em. ±	0.71	0.77	0.52	1.29	1.44	0.82	0.10	0.09	0.07
C.D. (P = 0.05)	2.08	2.27	1.51	3.79	4.23	2.40	0.29	0.25	0.21
Planting geometry (P)									
P_1–_30 cm x 10 cm	26.54	26.48	26.51	49.21	49.21	49.21	2.94	2.95	2.95
P_2_ **-** 22.5 cm x 10 cm	26.59	27.06	26.83	50.23	50.74	50.49	3.04	3.04	3.04
S. Em. ±	0.58	0.63	0.42	1.05	1.18	0.67	0.08	0.07	0.06
C.D. (P = 0.05)	NS	NS	NS	NS	NS	NS	NS	NS	NS
Harvesting stages (H)									
H_1_ **-** Earhead emergence	24.53	24.48	24.50	46.21	46.74	46.47	2.45	2.45	2.45
H_2_ **-** Physiological maturity	28.61	29.06	28.83	53.23	53.21	53.22	3.53	3.54	3.54
S. Em. ±	0.58	0.63	0.42	1.05	1.18	0.67	0.08	0.07	0.06
C.D. (P = 0.05)	1.70	1.85	1.23	3.09	3.45	1.96	0.23	0.21	0.17
Interaction (N x P)									
S. Em. ±	0.85	0.87	0.86	1.60	1.61	1.61	0.09	0.08	0.09
C.D. (P = 0.05)	NS	NS	NS	NS	NS	NS	NS	NS	NS
Interaction (N x H)									
S. Em. ±	0.85	0.87	0.86	1.60	1.61	1.61	0.09	0.08	0.09
C.D. (P = 0.05)	NS	NS	NS	NS	NS	NS	NS	NS	NS
Interaction (P x H)									
S. Em. ±	0.70	0.72	0.70	1.30	1.32	1.31	0.08	0.07	0.08
C.D. (P = 0.05)	NS	NS	NS	NS	NS	NS	NS	NS	NS
Interaction (N x P x H)									
S. Em. ±	1.42	1.55	1.03	2.23	2.89	2.28	0.19	0.17	0.14
C.D. (P = 0.05)	NS	NS	NS	NS	NS	NS	NS	NS	NS

Planting geometry did not significantly affect ADF, NDF or lignin content during either year or in the pooled analysis (**Table 5**). Comparable values of fiber fractions were recorded under both spacing treatments, indicating that variation in plant density within the tested range had minimal influence on the structural composition of the forage. This suggests that forage quality in terms of fiber fractions was primarily governed by nutrient supply and harvest stage rather than spatial arrangement of plants.

Harvesting stage had a pronounced effect on all quality parameters. Harvesting at earhead emergence (H_1_) resulted in significantly lower pooled ADF (24.50%), NDF (46.47%) and lignin (2.45%) contents compared with harvesting at physiological maturity (H_2_), which recorded pooled values of 28.83%, 53.22% and 3.54%, respectively. The increase in fiber and lignin content at physiological maturity can be attributed to advanced plant development, characterized by increased cell wall thickening, lignification and accumulation of structural carbohydrates. Consequently, harvesting at earlier stages favored superior forage quality, while delayed harvesting resulted in increased fiber concentration and reduced digestibility.

The interaction among nitrogen levels and planting geometry, nitrogen levels and harvesting stages, planting geometry and harvesting stages and nitrogen levels, planting geometry and harvesting stages (N × P × H) was not significant for ADF, NDF and lignin during both years and in pooled analysis, indicating that the individual effects of nitrogen and harvesting stage on forage quality were consistent across planting geometries. The present findings are in close agreement with earlier reports of Mohan et al. (2017) in teosinte, Shekara et al. (2025) in fodder maize, Luo et al. (2019) and Jagadeesh et al. (2017) in napier grass.

#### 3.4.3 Total digestible nutrients, dry matter digestibility, nitrogen-free extract and cell contents

Nitrogen levels significantly influenced forage quality parameters, including total digestible nutrients (TDN), dry matter digestibility (DMD) and nitrogen-free extract (NFE) during both years of study and in the pooled analysis (**Table 6**). Increasing nitrogen application resulted in a marked improvement in digestibility-related traits. Application of 80 kg N ha^-1^ (N_3_) recorded the highest pooled TDN (69.90%) and DMD (70.64%), which were significantly superior to those obtained with 60 kg N ha^-1^ (66.66% and 68.06%, respectively) and 40 kg N ha^−1^ (63.88% and 65.79%, respectively). In contrast, pooled NFE content declined with increasing nitrogen level, with the highest value recorded under 40 kg N ha^−1^ (52.18%) and comparatively lower values under 60 and 80 kg N ha^-1^. The improvement in TDN and DMD at higher nitrogen levels can be attributed to enhanced forage quality through reduced fiber fractions and increased availability of digestible nutrients, while the decline in NFE suggests a shift in assimilate partitioning towards protein and structural components.

**Table 6:** Effect of nitrogen levels, planting geometry and harvesting stages on TDN, DMD, NFE and cell content of barnyard millet fodder (grouped data of two years).

Treatment	TDN			DMD			NFE		
	2024	2025	Pooled	2024	2025	Pooled	2024	2025	Pooled
Nitrogen levels (N)									
N_1–_40 kg N ha^-1^	64.13	63.63	63.88	65.96	65.73	65.79	50.99	50.63	52.18
N_2–_60 kg N ha^-1^	67.05	66.26	66.66	68.16	67.87	68.06	49.82	49.43	48.56
N_3–_80 kg N ha^-1^	69.96	69.83	69.90	70.17	70.50	70.64	47.74	52.22	49.75
S. Em. ±	0.91	1.12	0.96	0.92	1.03	1.05	0.57	0.32	0.97
C.D. (P = 0.05)	2.68	3.28	2.83	2.71	3.04	3.08	1.68	0.94	2.85
Planting geometry (P)									
P_1–_30 cm x 10 cm	67.08	66.25	66.67	68.15	68.25	68.27	48.91	50.99	49.97
P_2_ **-** 22.5 cm x 10 cm	67.02	66.89	66.96	68.04	67.81	68.05	50.12	50.53	50.36
S. Em. ±	0.75	0.91	0.79	0.92	1.03	1.05	0.47	0.26	0.79
C.D. (P = 0.05)	2.19	2.68	2.31	2.71	3.04	3.08	1.37	0.77	2.33
Harvesting stages (H)									
H_1_ **-** Earhead emergence	69.68	68.77	69.22	69.76	69.82	69.82	48.80	51.25	48.90
H_2_**-**Physiological maturity	64.42	64.38	64.40	66.43	66.24	66.51	50.23	50.27	51.44
S. Em. ±	0.75	0.91	0.79	0.92	1.03	1.05	0.47	0.26	0.79
C.D. (P = 0.05)	2.19	2.68	2.31	2.71	3.04	3.08	1.37	0.77	2.33
Interaction (N x P)									
S. Em. ±	1.05	1.24	1.13	1.15	1.37	1.24	1.00	0.93	0.97
C.D. (P = 0.05)	NS	NS	NS	NS	NS	NS	NS	NS	NS
Interaction (N x H)									
S. Em. ±	1.05	1.24	1.13	1.15	1.37	1.24	1.00	0.93	0.97
C.D. (P = 0.05)	NS	NS	NS	NS	NS	NS	NS	NS	NS
Interaction (P x H)									
S. Em. ±	0.96	1.04	0.99	1.07	1.16	1.12	0.76	0.60	0.67
C.D. (P = 0.05)	NS	NS	NS	NS	NS	NS	NS	NS	NS
Interaction (N x P x H)									
S. Em. ±	1.83	2.24	1.93	2.26	2.53	2.57	1.14	0.64	1.94
C.D. (P = 0.05)	NS	NS	NS	NS	NS	NS	NS	NS	NS

Planting geometry did not exert a significant effect on pooled TDN, DMD or NFE (**Table 6**). Although minor variations were observed between years, the overall similarity of values under both spacing treatments indicates that plant density within the tested range had limited influence on digestibility and carbohydrate fractions of the forage (Sher et al., 2017).

Harvesting stage significantly affected all three quality parameters. Harvesting at earhead emergence (H_1_) resulted in significantly higher pooled TDN (69.22%) and DMD (69.82%) compared with harvesting at physiological maturity (H_2_), which recorded pooled values of 64.40% and 66.51%, respectively. Conversely, pooled NFE content was significantly higher at physiological maturity (51.44%) than at earhead emergence (48.90%). The reduction in digestibility parameters at later stages of harvest may be attributed to increased lignification and accumulation of structural carbohydrates, whereas the increase in NFE at physiological maturity reflects greater accumulation of non-structural carbohydrates as the crop advanced towards maturity.

The interaction among nitrogen levels and planting geometry, nitrogen levels and harvesting stages, planting geometry and harvesting stages and nitrogen levels, planting geometry and harvesting stages (N × P × H) was not significant for TDN, DMD and NFE during both years and in the pooled analysis, indicating that the effects of individual management factors on forage quality parameters were largely independent.

#### 3.4.4 Correlation coefficients for dry fodder yield and forage quality parameters

The correlation analysis among dry fodder yield and major nutritional quality attributes of barnyard millet revealed several strong and biologically meaningful relationships (**Table 7**). Dry fodder yield exhibited positive and moderate correlations with ash (r = 0.758), cell content (r = 0.517), DMD (r = 0.507) and TDN (r = 0.438), indicating that improvements in yield were generally associated with enhanced digestibility and mineral content of the fodder. Conversely, dry fodder yield was negatively correlated with fiber fractions such as ADF (r = –0.362), NDF (r = –0.307), crude fiber (r = –0.317), lignin (r = –0.181) and NFE (r = –0.443). This trend is consistent with forage quality principles where higher fiber fractions reduce digestibility and ultimately biomass conversion efficiency.

**Table 7:** Matrix of correlation coefficients for dry fodder yield and forage quality parameters in barnyard millet (grouped data of two years).

	Dry fodder yield	Crude fibre	Crude protein	Ether extractable fat	ASH	ADF	NDF	Lignin	TDN	DMD	NFE	Cell content
DRY FODDER YIELD	1											
CRUDE FIBRE	-0.3165	1										
CRUDE PROTEIN	0.031738	-0.87245	1									
ETHER EXTRACTABLE FAT	0.21562	-0.91063	0.847442	1								
ASH	0.758174	-0.79374	0.61479	0.641712	1							
ADF	-0.36228	0.910441	-0.87699	-0.91495	-0.76938	1						
NDF	-0.30723	0.971916	-0.90678	-0.94218	-0.76829	0.95328	1					
LIGNIN	-0.18138	0.843917	-0.84415	-0.77102	-0.60117	0.888385	0.860946	1				
TDN	0.438278	-0.87165	0.801223	0.893321	0.810507	-0.96922	-0.916	-0.81393	1			
DMD	0.506566	-0.91283	0.799696	0.906988	0.84015	-0.98154	-0.94485	-0.83259	0.973044	1		
NFE	-0.44279	0.711562	-0.77409	-0.551	-0.81276	0.796988	0.753153	0.739725	-0.75307	-0.77314	1	
CELL CONTENT	0.51733	-0.91445	0.727693	0.884441	0.816139	-0.88067	-0.94194	-0.7278	0.867362	0.931833	-0.6802	1

Crude fiber showed strong negative correlations with crude protein (r = –0.872), ether extract (r = –0.911), TDN (r = –0.872) and DMD (r = –0.913), reaffirming that increased structural carbohydrates depress both nutritive value and digestible nutrient availability. Fiber fractions (ADF and NDF) exhibited very strong positive correlations with crude fiber (r = 0.910 and 0.972, respectively) and lignin (r = 0.888 and 0.861), but strong negative correlations with crude protein (r = –0.877 and –0.907), TDN (r = –0.969 and –0.916), and DMD (r = –0.982 and –0.945). This highlights the central role of cell wall components in determining forage digestibility.

Crude protein maintained positive associations with ether extract (r = 0.847), TDN (r = 0.801) and DMD (r = 0.800), suggesting that protein-rich samples tended to be more digestible and energy-dense. Ash content showed moderate-to-strong positive correlations with DMD (r = 0.840), TDN (r = 0.811) and cell content (r = 0.816), indicating that mineral-rich fodder also supported better nutrient availability. Ether extractable fat, a key contributor to energy density, demonstrated strong positive correlations with TDN (r = 0.893), DMD (r = 0.907), and cell content (r = 0.884), confirming its role in enhancing digestibility.

Lignin, a major indigestible component, was negatively correlated with crude protein (r = –0.844), TDN (r = –0.814), DMD (r = –0.833) and cell content (r = –0.728), reflecting its detrimental effect on forage quality. NFE displayed negative correlations with most digestibility parameters, including TDN (r = –0.753) and DMD (r = –0.773), underscoring that excessive non-fibrous carbohydrate does not necessarily enhance quality when associated with lower protein and higher structural components.

Overall, the correlation structure clearly indicates that fiber fractions (ADF, NDF, lignin) are the primary determinants limiting forage quality, while crude protein, ash, ether extract and cell content strongly contribute to improved digestibility and energy value. The observed relationships align with established forage quality models, confirming that barnyard millet follows typical C4 forage behavior where increasing structural carbohydrates compromise both quality and digestible nutrient yield.

## 4 Conclusion

The present study demonstrated that barnyard millet is a highly promising climate-resilient fodder crop capable of addressing fodder scarcity in dryland and resource-constrained regions. Among the evaluated treatments, nitrogen application of 80 kg N ha^−1^ consistently produced superior green and dry fodder yields, improved nutrient uptake and enhanced overall forage nutritive quality. Closer planting geometry (22.5 × 10 cm) proved beneficial in maximizing biomass production without compromising quality, indicating better canopy coverage and efficient use of available resources. Harvesting at the earhead emergence stage significantly improved crude protein, digestibility (TDN and DMD), and reduced fiber fractions (ADF, NDF, lignin), confirming that early cutting ensures higher-quality fodder, while harvesting at physiological maturity enhances dry matter accumulation. The absence of strong interaction effects suggests that nitrogen level, planting geometry and harvest timing independently influence fodder yield and quality. Correlation analysis further highlighted that fiber components (ADF, NDF and lignin) are the major factors limiting digestibility, whereas crude protein, ash and cell contents positively contribute to improved forage value. Overall, the study indicates that adopting 80 kg N ha^−1^ and closer spacing provides an effective management strategy for achieving high yield, superior nutritive value and improved nutrient uptake in barnyard millet. These findings offer practical guidance for farmers and fodder-based production systems aiming to enhance livestock productivity and ensure sustainable fodder security in semi-arid and dryland areas.

## Data Availability

The original contributions presented in the study are included in the article/supplementary material. Further inquiries can be directed to the corresponding author.
